# The Foreign Journals: Midwifery

**Published:** 1839-07

**Authors:** 


### MIDWIFERY.

### *On the Causes of Face Presentations, &c.* By Dr. J. F. Osiander, Professor of Medicine at Göttingen.

This Essay contains but little practical information respecting face presentations, as the groundwork upon which that information must be based, viz. a minute description of the manner in which the face presents and passes through the pelvis and external passages, is very defective. The author does not agree with Boer that the chin is invariably directed forwards, although this opinion was confirmed by Siebold, who declared, that he had never met with a face presentation where the chin was turned towards the sacrum, an opinion also confirmed by Naegele, &c. He remarks, that it is scarcely credible how much the different parts of the face are squeezed together during labour, a circumstance in which we fully agree, having, on more than one occasion, had the opportunity of *seeing* the face as it was passing over the perineum. In speaking of the relative frequency of face cases, he shows an apparent discrepancy in the experience of different authors: thus La Motte, out of 400 labours, observed only two presentations of the face; Mauriceau, in 700 cases, only four; Giffard only one case in 200; Portal three in 80, and Smellie nine: whereas Boer is stated to have observed 243 cases of face presentation in seventeen years, of which only three cases required artificial assistance; the correctness of this statement Dr. Osiander considers invalidated by the manner in which the reports of the Vienna Lying-in Hospital were kept; a circumstance which we should be inclined to doubt, when we consider the great extent of this establishment, and the high character of the celebrated Boer. The author gives a long list of causes; all of which, with the exception of an unusual quantity of liquor amnii, are in our opinion merely imaginary.

*Hamburg. Zeitschrift. December,* 1838.

